# Heat stress exposure cause alterations in intestinal microbiota, transcriptome, and metabolome of broilers

**DOI:** 10.3389/fmicb.2023.1244004

**Published:** 2023-09-19

**Authors:** Xuan Liu, Zhenhua Ma, Yanfei Wang, Hao Jia, Zheng Wang, Lihuan Zhang

**Affiliations:** Shanxi Key Lab. for the Modernization of TCVM, College of Life and Science, Shanxi Agricultural University, Taigu, China

**Keywords:** heat stress, microbiota, transcriptome, metabolome, broiler

## Abstract

**Introduction:**

Heat stress can affect the production of poultry through complex interactions between genes, metabolites and microorganisms. At present, it is unclear how heat stress affects genetic, metabolic and microbial changes in poultry, as well as the complex interactions between them.

**Methods:**

Thus, at 28  days of age a total of 200 Arbor Acres broilers with similar body weights were randomly divided into the control (CON) and heat stress treatment (HS). There were 5 replicates in CON and HS, respectively, 20 per replication. From the 28–42  days, the HS was kept at 31 ± 1°C (9:00–17:00, 8 h) and other time was maintained at 21 ± 1°C as in the CON. At the 42nd day experiment, we calculated the growth performance (*n* = 8) of broilers and collected 3 and 6 cecal tissues for transcriptomic and metabolomic investigation and 4 cecal contents for metagenomic investigation of each treatment.

**Results and discussion:**

The results indicate that heat stress significantly reduced the average daily gain and body weight of broilers (value of *p* < 0.05). Transcriptome KEGG enrichment showed that the differential genes were mainly enriched in the NF-kB signaling pathway. Metabolomics results showed that KEGG enrichment showed that the differential metabolites were mainly enriched in the mTOR signaling pathway. 16S rDNA amplicon sequencing results indicated that heat stress increased the relative abundance of *Proteobacteria* decreased the relative abundance of *Firmicutes*. Multi-omics analysis showed that the co-participating pathway of differential genes, metabolites and microorganisms KEGG enrichment was purine metabolism. Pearson correlation analysis found that ornithine was positively correlated with *SULT1C3*, *GSTT1L* and *g_Lactobacillus*, and negatively correlated with *CALB1*. PE was negatively correlated with *CALB1* and *CHAC1*, and positively with *g_Alistipes*. In conclusion, heat stress can generate large amounts of reactive oxygen and increase the types of harmful bacteria, reduce intestinal nutrient absorption and antioxidant capacity, and thereby damage intestinal health and immune function, and reduce growth performance indicators. This biological process is manifested in the complex regulation, providing a foundational theoretical basis for solving the problem of heat stress.

## 1. Introduction

Chicken is the second largest type of meat product in China, except for pork. The annual sales of broilers in China can reach 10.5 billion, and the per capita consumption has been increasing year by year. Compared to laying hens, broilers can provide humans with more high-quality dietary protein and meat products (Resnyk et al., 2017). Heat stress is one of the biggest challenges facing global animal husbandry. The increase in ambient temperature and humidity will affect the production of animals in summer and will cause economic losses in severe cases (Salem et al., 2022). Heat stress causes the high mortality rate and severe economic losses estimated at 240 million United States dollars per year in the poultry sector (St-Pierre et al., 2003), representing, for example, about 7% of total HS-caused losses in the French livestock industry in 2003 (Nardone et al., 2010). According to the time of heat stress, it can be divided into acute heat stress (<7 days) and chronic heat stress (≥7 days); According to the temperature of stress treatment, it can be divided into cyclic heat stress and sustained heat stress (Gonzalez-Esquerra and Leeson, 2006). Research has shown that acute heat stress can cause a heat shock response, leading to rapid initiation of heat shock protein synthesis and rapid changes in gene expression, while chronic heat stress can cause larger scale changes (Xie et al., 2014). In addition, Xie et al. (2015) reported that chronic heat stress can lead to tissue damage. Research has found that the cyclic heat stress pattern is more in line with the characteristics of summer temperature and closer to the actual living environment (Al Qaisi et al., 2022). Heat stress can affect intestinal microflora’s activity and stimulate the hypothalamus’s feeding center to reduce its excitability, leading to the decline of broiler growth performance and even death (Wang, 2013). In addition, broilers will respond to changes in the external environment by adjusting their metabolic levels, which can detect changes in gene, metabolite and microorganism levels through behavioral changes and the use of transcriptome, metabolome and 16S rDNA amplicon sequencing technology (Jastrebski et al., 2017).

It has revealed the influence of heat stress on broilers’ cecum by the transcriptome, metabolome and 16S rDNA amplicon technology. Transcriptome sequencing (RNA-Seq) reflects the expression of a specific cell or tissue gene and has the advantage of high sensitivity. RNA-Seq has been successfully applied to study the molecular basis of complex traits, such as feed efficiency and myopathy, and evaluate the molecular response to nutritional therapy and important aspects of immunity and disease resistance (Zampiga et al., 2018). In addition, in terms of heat stress, RNA-Seq analysis was used to identify key genes that respond to the heat stress induced volatilization in laying hens, such as *PDK4* and *FGA* (Wang et al., 2021). Transcriptome analysis identified potential target genes, especially those involved in cell migration and immune signaling responses to heat stress, which can inform future research on heat stress in broilers and could prove useful for improving disease resistance (Monson et al., 2018). Metabolomics is a sequencing technology that studies the body’s physiological changes and pathological characteristics from a dynamic perspective through the qualitative and quantitative determination of metabolites (Nicholson et al., 1999). It can build a direct correlation between metabolites and biological phenotypes, dynamically track and analyze the metabolites of animal bodies, help to analyze the relationship between phenotypes and genetics, environmental and other factors, and provide a basis for the improvement and breeding of economic traits. Metabolomics is widely used to understand the changes in organism metabolism, such as metabolism, sugar, lipid, amino acid metabolism, and meat quality evaluation (Zhang Y. et al., 2023; Zhang F. et al., 2023; Zhang X. et al., 2023). Zampiga et al. (2021) found that chronic heat stress could regulate the changes of metabolites in breast muscle and plasma of broilers by metabolome, and found metabolites that might play an important role in regulating Energy homeostasis of the body. According to metabolomic analysis, heat stress has caused to changes in serum lipid metabolism of broilers. It is determined that heat stress can reduce the content of lysophosphatidylcholine and increase the content of phosphatidylcholine (Guo et al., 2021a). The 16S rDNA amplicon sequencing technology can analyze microbial communities to obtain information on community structure, differences and functions. It is also widely used in the poultry industry, revealing the interaction between microbial communities such as the gut, animal reproduction, growth and development, nutritional health, environmental factors, immunity and disease treatment (Pan and Yu, 2014). Emami et al. (2022) reported that using microbial amplicon analysis, it was found that heat stress can affect the diversity and composition of gut microbiota in broilers, which can provide a basis for developing nutritional strategies to maintain gut microbiota balance and alleviate the negative effects of heat stress on performance and health of broilers. In addition, the multi-omics analysis has shown unique advantages in revealing the underlying molecular regulatory mechanisms. For example, integrated analysis of transcriptome and metabolome was used to determine the critical amino acid metabolic pathway to improve duck eggs (Yan et al., 2023) and lipopolysaccharides could induce the immune stress pathway in broilers (Bi et al., 2022). Hubbard et al. (2019) reported that the combination of RNA-Seq and metabolomic data can identify new changes in gene regulation of broilers affected by heat stress, which reflect changes in pathways that affect metabolite levels. Integrated analysis of metabolome and microbiome has determined that heat stress can increase the relative abundance of harmful microbes in the cecum of broilers and reduce health-related metabolites such as L-malic acid, which can provide a basis for the impact of heat stress on physiological changes and intestinal health in broilers (Liu et al., 2022, 2022a,b).

The intestine is a sensitive part of heat stress and an important organ for nutrient absorption and immune regulation in poultry, making it crucial for poultry production (Deng et al., 2023). The cecum is the broiler gastrointestinal tract’s most diverse area of microbes and various microbes are crucial in improving growth performance and maintaining physical health (Luo et al., 2021). Previous studies mainly explored the effects of heat stress on broiler intestines from a single omics perspective, including transcriptome, metabolomics, and microbiome (Dridi et al., 2022; Kim et al., 2022; Zhang Y. et al., 2023; Zhang F. et al., 2023; Zhang X. et al., 2023). However, very few studies have comprehensively revealed the heat-stress impact on broilers’ guts. Therefore, this experiment conducted chronic heat stress treatment on broilers and analyzed the effects of heat stress on the cecum of broilers at the genetic, metabolic, and microbial levels to provide a theoretical basis for subsequent research.

## 2. Materials and methods

### 2.1. Animal ethics

The animal use protocol has been reviewed and approved by the Institutional Animal Care and Use Committee of Shanxi Agricultural University. All procedures involving the handling, management, and healthcare of live poultry follow the regulations for the use of experimental animals for scientific purposes, and are implemented in accordance with the Shanxi Agricultural University Ethics Committee (SXAU-EAW-2021C0630) in China.

### 2.2. Experiment design and animal feeding

Three hundred 1-day-old Arbor Acres broilers were purchased from Xiangfeng Poultry industry, Taigu county, Shanxi province. On the 28th day, 200 broilers with similar weights were selected and randomly divided into a control group (CON) and a heat stress treatment group (HS). There were 5 replicates in each group and had 20 broilers of each replication. All broilers were raised in a three-layer vertical cage, vaccinated as required, and regularly cleaned and disinfected the chicken house. The feeding period was divided into 1–21 days and 22–42 days. The trial period was 14 days. Feeding at 8:00 and 19:00 every day and *ad libitum* access to water and eating, the broilers were exposed to light for 23 h and dark for 1 h every day, and the temperature of the brood was 34 ± 1°C at 1–3 days, 32 ± 1°C at 4–7 days. After that, it declined by 1°C every day until it was kept at a constant temperature of 21 ± 1°C. On the 28th day, the broilers in the HS were subjected to chronic heat stress treatment until the end of the test. That was, the feeding temperature was raised to 33 ± 1°C at 9:00–17:00 daily, the heat stress treatment lasted for 8 h, and the rest of the time was kept at the same temperature as the control group (21 ± 1°C). The experimental broilers were fed and managed by the national standard GB/T 19664–2005 production technique criterion for commercial broiler. Each group was fed with corn-soybean meal basal diet. The corn-soybean meal formula was prepared according to the National Research Council recommendations. Analyzed nutrient concentrations in the experimental diets are reported in Supplementary Table S1.

### 2.3. Growth performance and sample collection

Prior to slaughter, broilers were prohibited from eating for 10 h and their body weight was recorded. Recorded the eating feed of broilers on time and calculated the average daily feed intake (ADFI), average daily gain (ADG), and feed conversion rate (FCR = ADFI/ADG) of each group of broilers at the end of the experiment. The original data of growth performance in Supplementary Table S2.

We took cecal samples from each group and washed with pre-cooled saline physiological solution. Then we quickly cooled them in liquid nitrogen, stored at −80°C fridge, and shipped three and six 2 cm^2^ cecal samples to Shanghai Meiji Biomedical Technology Co., Ltd. for transcriptome and metabolome sequencing, respectively. In addition, four 2 cm^2^ samples of cecal contents were also taken from each group and sent to Novogene Co., Ltd. for 16S rDNA amplicon sequencing.

### 2.4. Transcriptome analysis

#### 2.4.1. Extraction of total RNA

Trizol reagent was used to extract caecum tissue. The Nanodrop 2000 and Agilent 2100 bioanalyzers then detected total RNA, purity and RIN (RNA integrity number).

#### 2.4.2. Construction of the cDNA library

Took 1 μg of qualified total RNA sample, enriched the mRNA with magnetic beads, broke it up into 300 bp fragments, reversed transcribed the mRNA fragments into cDNA, and synthesized double-stranded cDNA. Added End Repair Mix to complement the end of the double-stranded cDNA from the sticky end to the flat end, added a tail at the 3′ end for ligating the adapter and obtained the final cDNA library after PCR amplification and purification. And then the cDNA library had been sequenced by the Illumina Novaseq 6000 platform.

#### 2.4.3. Alignment with the reference genome

In order to ensure data quality, the original data was filtered before analysis, and the low-quality data was filtered out to reduce the interference caused by invalid data to obtain clean reads. The quality-controlled clean reads were compared with the Ensemble reference genome (reference genome version: GRCg6a)^1^ to obtain mapped reads for subsequent transcript assembly and expression calculation. In the quantitative analysis of genes using the REEM software, the quantitative indicator was TPM (Transcripts per kilobase million).

#### 2.4.4. Gene clustering analysis and screening of differential genes

Genes were clustered to observe the effect of heat stress on broiler cecum genes. DESeq2 (Version 1.24.0) software was used for gene expression differential analysis to |Log2 Foldchange| ≥ 1 and *P*adjust < 0.05 were used as criteria for screening for differential genes. The multiple test correction method is BH. The detailed information of differential genes can be seen in Supplementary Table S3.

#### 2.4.5. Differential genes function enrichment

In order to analyze the function of differentially expressed genes, we conducted GO and KEGG functional enrichment analysis on differentially expressed genes using Goatools (Version 0.6.5) and R (Version 1.6.2). *P*adjust < 0.05 was used to evaluate significant enrichment of the GO function and KEGG enrichment analysis. The multiple test correction method is BH.

#### 2.4.6. Real-time PCR verification

Four differential genes (*PDK4*, *CHAC1*, *EOMES* and *SULT1C3*) were randomly chosen for the validation tests. *β-actin* was selected as the reference gene. Using the Primer Premier 5.0 to design primers, the primer information is shown in Supplementary Table S4. The primers were synthesized by Sangon Biotech (Shanghai) Co., Ltd. The relative expression of the different genes between the groups was calculated by the 2^−∆∆CT^ method, and the GraphPad Prism 8 software was used to plot.

### 2.5. Metabolome analysis

#### 2.5.1. Sample pretreatment

Accurately weighed 50 mg of the cecal sample in a 2 mL centrifuge tube. Added 400 μl of methanol extract containing 0.02 mg/ml internal standard (2-Chloro-L-phenylalanine) and grinded at 50 Hz and −10°C for 6 min. Extracted at 5°C and 40 kHz for 30 min by ultrasound. Left a −20°C for 30 min. Centrifugation at 13,000 g, 4°C for 15 min, and sampling an equal amount of supernatant for machine analysis.

#### 2.5.2. LC–MS analysis process

The instrumental analysis platform used in this test was LC–MS (Thermo Scientific, UHPLC-Q Exactive HF-X), and the column was ACQUITY UPLC HSS T3 (100 mm × 2.1 mm i.d., 1.8 μm, Waters, Milford, United States), chromatographic separation conditions were column temperature 40°C, flow rate 0.40 ml/min, injection volume of 2 μl. The flow component A was 95% water +5% acetonitrile (containing 0.1% formic acid), and mobile phase B was 47.5% acetonitrile +47.5% isopropanol +5% water (containing 0.1% formic acid).

The primary mass spectrometry condition was that the sample was ionized by electrospray. That was, the positive mode spray voltage was 3,500 V, and the negative mode spray voltage was 2,800 V. The mass spectrometry signal was acquired in positive and negative ion scanning modes, and the scanning range was 70–1,050 m/z. Heating temperature 400°C, capillary temperature 320°C, sheath flow velocity 40 arb, auxiliary airflow rate 10 arb, S-Lens voltage 50 V. The resolution MS2 was 17,500, and the Full MS was 70,000.

#### 2.5.3. Data preprocessing

ProgenesisQi (Waters corporation, Milford, United States) software was used to identify and integrate peaks. The result was a data matrix that can be used for subsequent analysis. MS and MS/MS mass spectrometry information were then combined with metabolic databases HMDB and Metlin to match while identifying metabolites according to secondary mass spectrometry matching scores, normalizing and log-converting data to Log10.

#### 2.5.4. Sample variability comparative analysis

To analyze the differences between groups, we conducted principal component analysis (PCA) and partial least squares discrimination analysis (PLS-DA) analysis. PCA analysis used the data conversion type as unit variance conversion, with a confidence level of 0.95. PLS-DA analysis adopted the PLS-DA data conversion type of pareto conversion. The PLS-DA confidence level was 0.95, and the number of replacements was 200.

#### 2.5.5. Screening differential metabolites and KEGG enrichment

The metabolites obtained above were screened for differential metabolites by projecting VIP >1, and FDR < 0.05 as screening criteria. The software used was R (Version 1.6.2). Perform compound classification analysis on differential metabolites. To further clarify the functions of differential metabolites, they were compared to the KEGG database and significantly enriched metabolic pathways were screened by setting *P*adjust < 0.05 as the standard. The multiple test correction method is BH.

### 2.6. 16S rDNA amplicon analysis

#### 2.6.1. Microbial DNA extraction and 16S rDNA amplicon sequencing

Microbial genomic DNA was extracted by the CTAB method, and then the purity and concentration of extracted DNA were detected on 1% agarose gel. PCR amplification was performed using diluted genomic DNA as a template. The primers for the 16S V34 region were 341F (CCTAYGGRBGCACAG) and 806R (GGACTACNNGGTATCTAAT; Xiao et al., 2022). PCR products were detected by agarose gel electrophoresis with a concentration of 2%, and the target bands were recovered using the gel recovery kit provided by Qiagen Company. Using NEBNext^®^ Ultra™ IIDNA Library Prep Kit was used for library construction, and the constructed library was subjected to Qubit and qPCR quantification. After the library was qualified, used the NovaSeq 6000 for machine sequencing. After sequencing, used the DADA2 module in the QIIME2 software to denoise and filter out sequences with an abundance less than 5 in order to obtain the final ASVs (Amplicon sequence variables). Subsequently, the classify-sklearn module in QIIME2 software (qiime2-2020.6) was used to compare the obtained ASVs with the database to obtain species information for each ASV.

#### 2.6.2. Bioinformatics analysis

Used the QIIME2 software to calculate the Shannon, Simpson, Chao1, and ACE indexes and used the Simpson exponent as a reference to draw a rarefaction curve. The composition of microorganisms was presented using Venn plots, PCA plots, top 10 phylum horizontal species relative abundance histograms, and top 10 genus horizontal species relative abundance histograms. We used the LEfSe software and set a threshold of 4 (LDA = 4) for significant difference species analysis. In addition, to study the function of microorganisms, we conducted PICRUSt functional prediction analysis and compared it to the KEGG database. The above are all using software R (Version 3.5.3).

### 2.7. Multi-omics analysis

The Pearson correlation coefficient was used to calculate the correlation between differential genes and metabolites, differential genes and ASVs, and differential metabolites and ASVs to obtain the interaction relationship between genes, metabolites, and microorganisms. In addition, by conducting KEGG co-enrichment analysis on differential genes and metabolites, the function of genes and metabolites co-participating in the pathway can be clarified. By comparing the KEGG differential metabolic pathway predicted by PICRUST function with the KEGG pathway enriched by metabolomics, it was found that co-participating pathways can clarify the contribution of microorganisms to metabolic products.

### 2.8. Data analysis

Using the SPSS 26.0 software, the value of *p* of 42 days body weight is calculated by a mixed model, with the impact of replication as a random variable and grouping as a fixed factor to evaluate the impact of grouping on body weight. Excluded the impact of replication on selected experimental broilers through mixed model calculations, the independent sample t-test was used to evaluate the significance in the subsequent comparison between the two groups of data. The α diversity index was showed using GraphPad Prism 8 software. The value of *p* is obtained through calibration using the “BH” method. The value of *p* < 0.05 is considered statistically significant.

## 3. Results

### 3.1. Growth performance

Using the mixed model to evaluate the impact of replication on 42 days body weight by using replication as a random variable and grouping as a fixed effect. We obtained that replication did not have a significant impact on body weight (value of *p* > 0.05). Therefore, this eliminates the influence of random variables on the data. From Table 1, it can be seen that compared with the control group, the broilers of the HS showed a significant decrease in body weight and ADG at 42 days (value of *p* < 0.05), while there were no significant changes in ADFI and FCR (value of *p* > 0.05).

**Table 1:** Effects of heat stress on growth performance of broilers.

Index^1^	CON	HS	value of *p*
42d BW, g	2911.243 ± 7.076	2658.173 ± 14.016	0.001
ADG, g/bird	86.191 ± 1.991	73.547 ± 1.823	0.001
ADFI, g/bird	168.630 ± 6.037	151.144 ± 5.828	0.084
FCR, g/g	1.963 ± 0.079	2.071 ± 0.117	0.456

BW, body weight; ADG, average daily gain; ADFI, average daily feed intake; FCR, feed conversion rate.

1 CON = broilers were raised in an environment of 21 ± 1°C; HS = broilers were raised in an environment of 33 ± 1°C, treated with heat stress for 8 h per day, and maintained an appropriate temperature (21 ± 1°C) for the other time. All measurements were expressed as mean ± SEM (*n* = 8). The value of *p* of 42 days BW is calculated by a mixed model, with the impact of replication as a random variable and grouping as a fixed factor to evaluate the impact of grouping on body weight. The *p*-values of ADG, ADFI and FCR is calculated by the independent sample t-test. And all *p*-values have been corrected using the “BH” method.

### 3.2. Transcriptome results

#### 3.2.1. Quality control and reference genome comparison

Table 2 shows that the percentage of Q30 bases obtained by quality control is above 93.36%, Q20 is above 97.45, and the average error rate of sequencing bases is below 0.1%. The number of clean reads on the genome accounted for 88.78–90.53%, the alignment rate of multiple alignment positions on the reference sequence was 2.26–2.74%, and the alignment rate of the unique alignment position on the reference sequence was 86.24–87.88%. These results indicate that sequencing results had a low error rate and a high alignment rate of genes on the reference genome, which can be used for subsequent analysis.

**Table 2:** Sequencing data and alignment information.

Sample^1^	CON1	CON2	CON3	HS1	HS2	HS3
Group	Control	Control	Control	Heat stress	Heat stress	Heat stress
Raw reads	54,502,890	50,813,730	50,960,416	46,432,436	45,547,564	45,510,350
Raw bases, Gb	8,229,936,390	7,672,873,230	7,695,022,816	7,859,225,537	7,323,250,044	7,434,083,069
Clean reads	53,687,106	50,000,698	50,264,114	45,626,058	44,874,338	44,849,600
Clean bases, Gb	7,008,919,888	6,999,778,952	6,571,921,358	6,737,791,062	6,752,081,444	6,331,100,604
Q20, %	97.58	97.54	97.61	97.45	97.60	97.53
Q30, %	93.61	93.51	93.64	93.36	93.66	93.46
Error rate, %	0.0257	0.0258	0.0257	0.0258	0.0259	0.0256
Total mapped, %	89.20	89.65	90.22	88.78	90.53	90.51
Multiple mapped, %	2.26	2.66	2.34	2.53	2.74	2.72
Uniquely mapped, %	86.94	86.99	87.88	86.24	87.79	87.79

1 CON = broilers were raised in an environment of 21 ± 1°C; HS = broilers were raised in an environment of 33 ± 1°C, treated with heat stress for 8 h per day, and maintained an appropriate temperature (21 ± 1°C) for the other time.

#### 3.2.2. Gene clustering analysis and screening of differential genes

**Figure 1:**
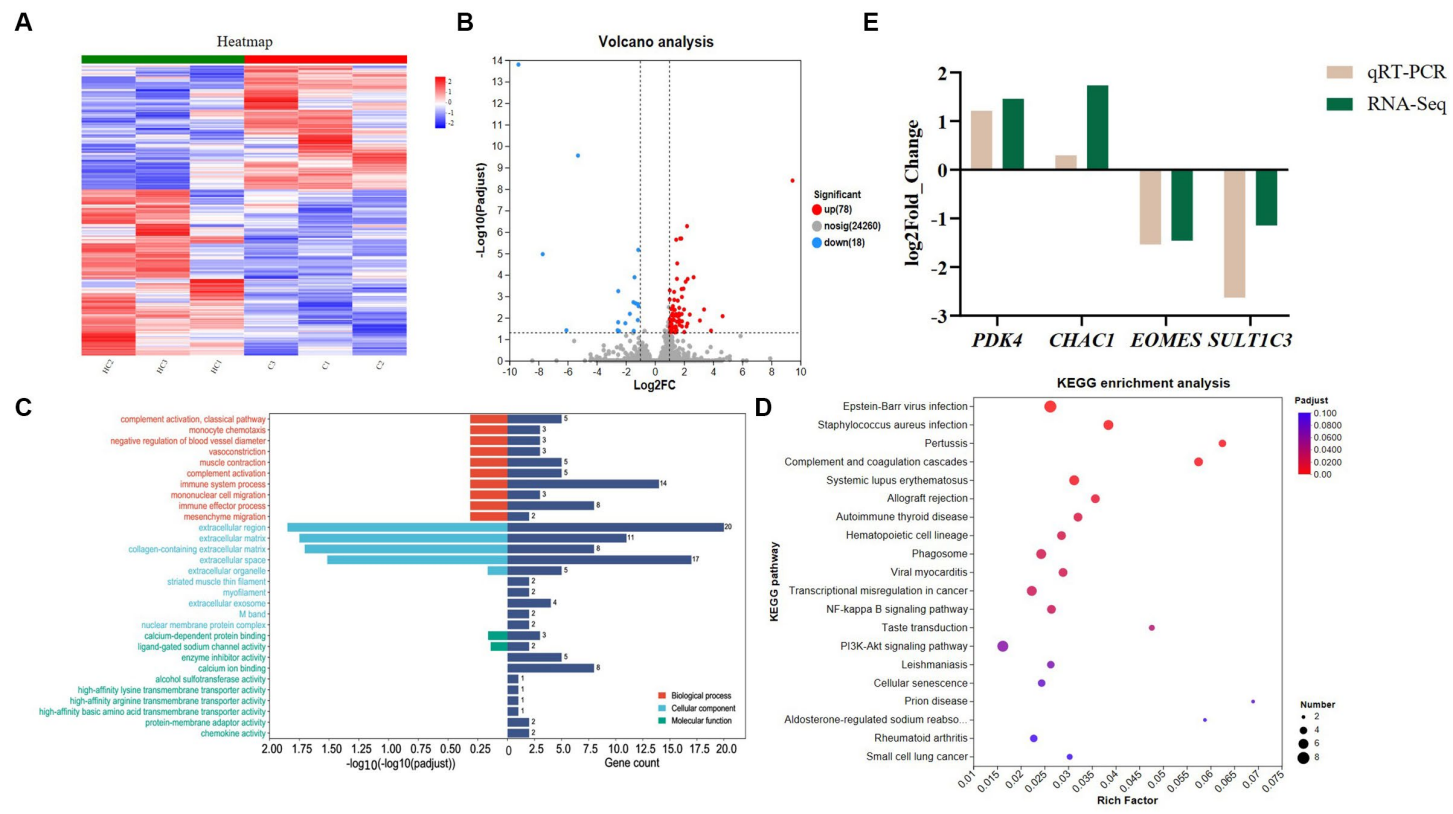
Transcriptome analysis and qPCR validation results. **(A)** Gene clustering heatmap, **(B)** volcano map analysis of differential genes, up, down and nosig stand for up-regulated, down-regulated and insignificant differentially insignificant genes, respectively. The criteria for screening differential genes are |Log2 Foldchange | ≥ 1 and *P*adjust < 0.05. **(C)** GO enrichment analysis, BP, CC and MF stand for biological process, cellular components and molecular function, respectively. The standard for screening GO items with differences is *P*adjust < 0.05. **(D)** KEGG enrichment analysis. The standard for screening KEGG pathways with differences is *P*adjust < 0.05. **(E)** verification of differential genes expression by qPCR. The cecal samples of 42-day-old broilers was collected for transcriptome analysis (*n* = 3). CON: broilers were raised in an environment of 21 ± 1°C. HS: broilers were raised in an environment of 33 ± 1°C (9:00–17:00) at 28 days, subjected to heat stress treatment for 8 h, and remained at an appropriate temperature (21 ± 1°C) for the other time as in CON.

From Figure 1A, it can be seen that there is a significant change in the gene expression level between the CON and HS. According to the screening criteria, we screened 96 differential genes, of which 78 were up-regulated and 18 were down-regulated (Figure 1B).

#### 3.2.3. Go enrichment analysis

We conducted GO enrichment analysis on differential genes. Figure 1C shows the top 30 GO metabolic entries (Supplementary Table S5). It can be seen from Figure 1C that the enrichment of differential genes in biological process (BP) mainly included immune system process, immune effector process and complement activation. The main activities involved in the enrichment of cellular components (CC) and molecular function (MF) were extracellular region, extracellular space, collagen-containing extracellular matrix, calcium-dependent protein binding and enzyme inhibitor activity.

#### 3.2.4. KEGG enrichment analysis

To further investigate the function of the differential genes, we conducted the KEGG enrichment analysis (Supplementary Table S6). Figure 1D shows the top 20 differential pathways. Among them, the NF-kB signaling pathway, and cellular senescence were related to regulating heat stress. In addition, although the enriched calcium signaling pathway and intestinal immune network for IgA production are not shown in Figure 1D, they are also enriched and crucial in regulating heat stress.

#### 3.2.5. qPCR verification

The results of Figure 1E show that the qPCR results are consistent with the trend of transcriptome results and have a high similarity, indicating that the transcriptome data are reliable and accurate.

### 3.3. Metabolome results

#### 3.3.1. PCA and PLS-DA analysis

**Figure 2:**
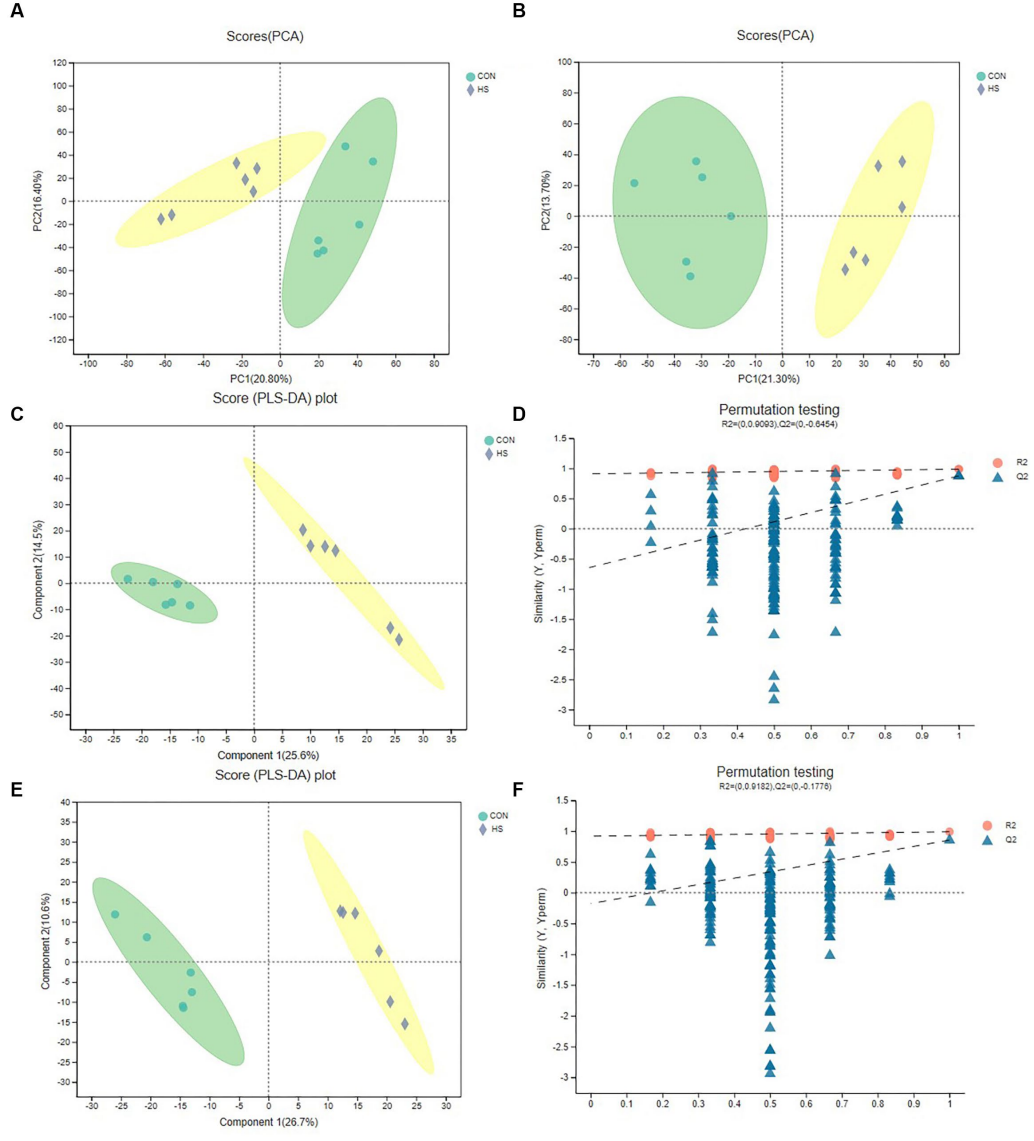
PCA and PLS-DA analysis of metabolomics. **(A)** Cation PCA diagram. **(B)** Anion PCA diagram. **(C)** Cation PLS-DA diagram. **(D)** Cation PLS-DA displacement test chart, the abscissa represents the displacement retention of the displacement test (the proportion consistent with the order of Y variables of the original model, the point with the displacement retention of 1 is the *R*^2^ and *Q*^2^ values of the original model), the ordinate represents the values of *R*^2^ (blue dot) and *Q*^2^ (red triangle) displacement test, and the two dashed lines represent the regression lines of *R*^2^ and *Q*^2^, respectively **(E)** Anion PLS-DA diagram. **(F)** Anion PLS-DA replacement test chart. The cecal samples of 42-day-old broilers was collected for metabolomic analysis (*n* = 6). CON: broilers were raised in an environment of 21 ± 1°C. HS: broilers were raised in an environment of 33 ± 1°C (9:00–17:00) at 28 days, subjected to heat stress treatment for 8 h, and remained at an appropriate temperature (21 ± 1°C) for the other time as in CON.

For the analysis of anions and cations, Figures 2A,B show the distribution of cations and anions between PCA analysis groups, respectively, and it could be found that the differences between groups were significant. Although PCA analysis can reveal differences between sample groups, this algorithm has limitations. Therefore, to further verify the differences between groups, we selected the PLS-DA test (Figures 2C–F). Figures 2C,E were the cation and anion distributions, respectively, and it could be found that the differences between the groups were evident. The distance of the samples in the group was closer. At the same time, the PLS-DA replacement test was carried out. And Figures 2D,F were, respectively, cation and anion replacement test results. The *R*^2^ and *Q*^2^ of the anion and cation were 0.9182, −0.1776, 0.9093 and −0.6454, respectively. Theoretically, the closer *R*^2^ and *Q*^2^ were to 1, the more stable the model, the better the prediction ability, and the high confidence of the results. The above results indicated that metabolite differences between were groups were significant.

#### 3.3.2. Screen for differential metabolites

**Figure 3:**
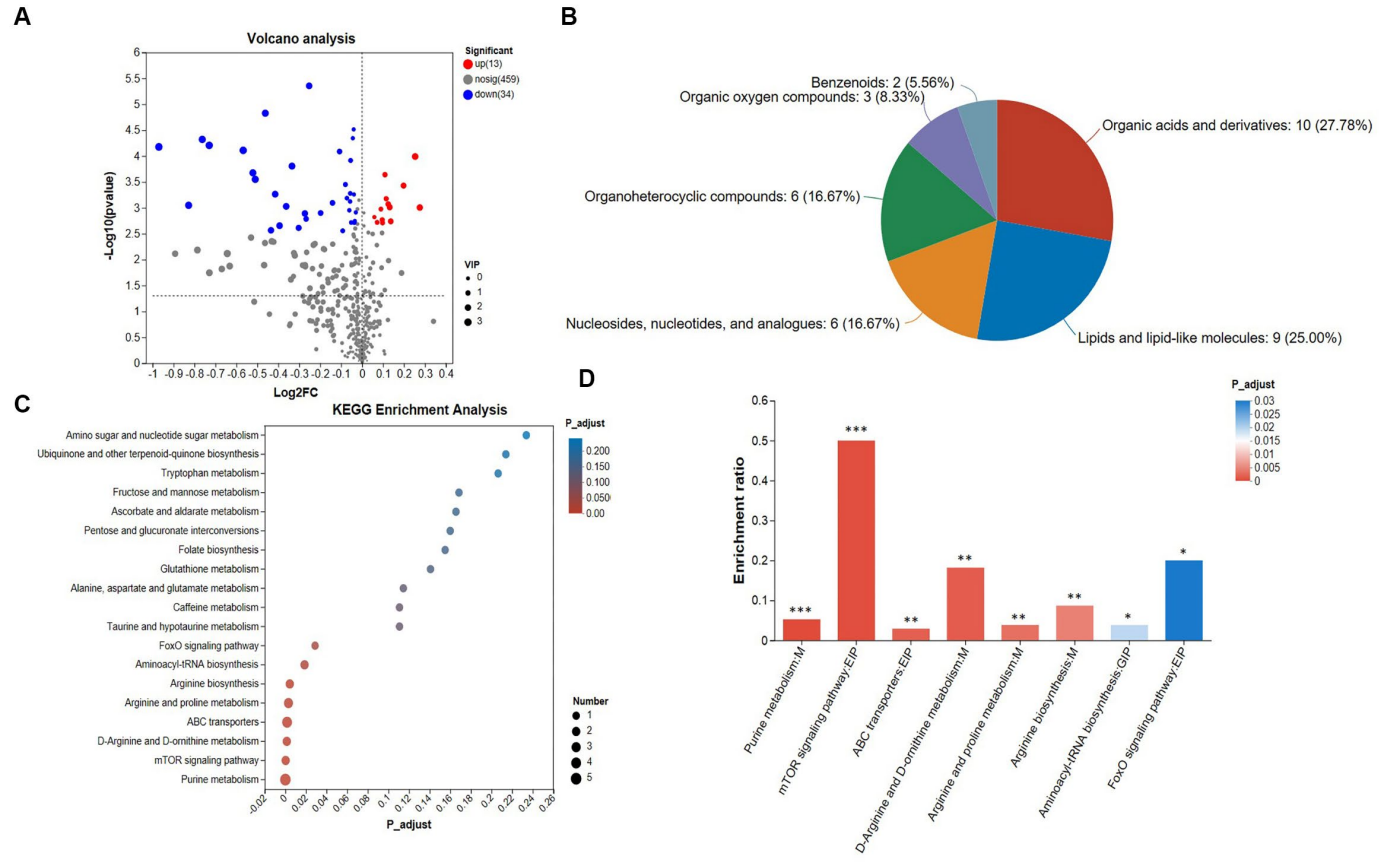
Analysis of metabolomics results. **(A)** Volcano plot. The criteria for screening differential metabolites are VIP > 1 and FDR < 0.05. **(B)** HMDB classification chart, **(C)** top 20 KEGG enrichment analysis. The standard for screening differential metabolic pathways is *P*adjust < 0.05. **(D)** Significant difference KEGG enrichment analysis. The standard for screening differential metabolic pathways is *P*adjust < 0.05. The cecal samples of 42-day-old broilers was collected for metabolomic analysis (*n* = 6).

Six hundred and nineteen differential metabolites were screened, of which 47 metabolites of known structures were screened (Supplementary Table S7). Figure 3A shows that 13 metabolites of known structures were up-regulated (4 and 9 anionic and cation metabolites, respectively), and 34 metabolites were down-regulated (19 and 15 anionic and cation metabolites, respectively) among the metabolites of known structures.

#### 3.3.3. Differential metabolic species class analysis

The superclass classification hierarchy classifies the distribution of differential metabolites in the HMDB database, and Figure 3B shows the HMDB classifications. Among them, organic acids and derivatives accounted for 27.78%, lipids and lipid-like molecules accounted for 25.00%, organoheterocyclic compounds accounted for 16.67%, nucleosides, nucleotides, and analogues accounted for 16.67%, organic oxygen compounds accounted for 8.33%, and benzenoids accounted for 5.66%.

#### 3.3.4. KEGG enrichment analysis

A total of 19 KEGG pathways are enriched (Supplementary Table S8; Figure 3C). We found that the number of metabolites enriched in the purine metabolism pathway was the largest. Further, we analyzed the pathways with significant differences. Eight differential pathways were screened for KEGG enrichment analysis of differential metabolites (Figure 3D), including the FoxO signaling pathway, mTOR signaling pathway, D-arginine and D-ornithine metabolism, arginine biosynthesis, aminoacyl-tRNA biosynthesis, arginine and proline metabolism, ABC transporters and purine metabolism pathway, all of which play a direct or indirect role in regulating heat stress.

### 3.4. 16S rDNA amplicon results

#### 3.4.1. α Diversity analysis

**Figure 4:**
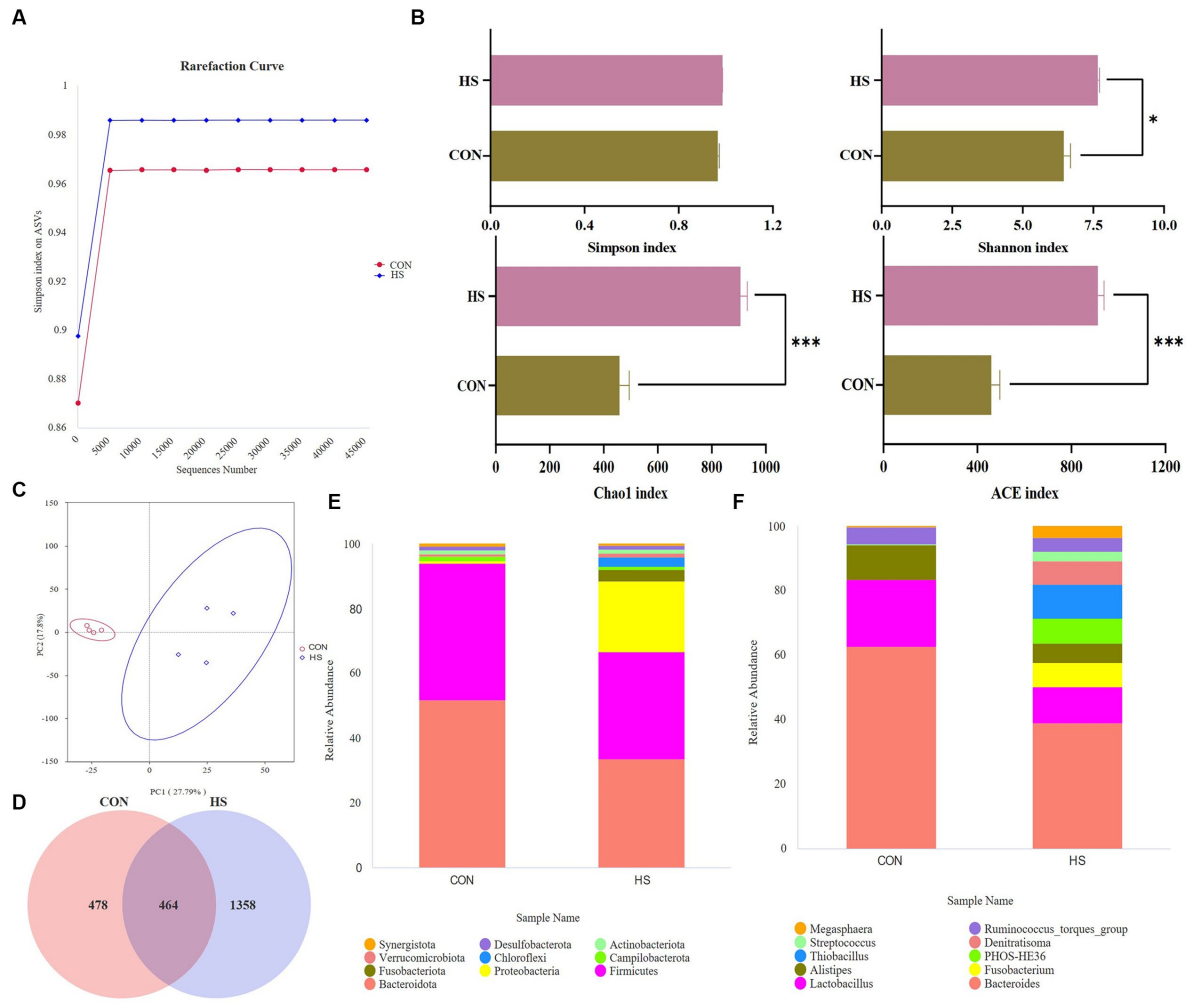
Analysis of microbial changes in the microbiome. **(A)** Rarefaction curve, **(B)** α diversity indexes, the screening criteria are value of *p* < 0.05. **(C)** PCA plot, **(D)** Venn plot, top 10 species at the **(E)** phylum level, and the relative abundance of the top 10 species at the **(F)** genus level. The cecal contents of 42-day-old broilers was collected for metabolomic analysis (*n* = 4). CON: broilers were raised in an environment of 21 ± 1°C. HS: broilers were raised in an environment of 33 ± 1°C (9:00–17:00) at 28 days, subjected to heat stress treatment for 8 h, and remained at an appropriate temperature (21 ± 1°C) for the other time as in CON.

Figure 4B shows that heat stress increases the abundance of microbial communities and the types of low-abundance species. Randomly selected a certain amount of data from the sample and calculated the rarefaction curve based on the Simpson index. As shown in Figure 4A, as the curve tends to flatten out, the sequencing data volume is more reasonable, and more data volume will not affect α diversity index has a significant impact.

#### 3.4.2. Microbial composition analysis

The principal component analysis (PCA) showed a variation of 27.79% for PC1 and 17.8% for PC2 (Figure 4C). After filtering out the low-quality data, 478 and 1,358 ASVs were observed in CON and HS, respectively, and 464 ASVs in both groups (Figure 4D). Analyzing their species, *Bacteroidota*, *Firmicutes*, and *Proteobacteria* were the dominant species in the cecum of 42-day-old broilers. Meanwhile, heat stress increased the relative abundance of *Proteobacteria* in broilers and decreased the relative abundance of *Bacteroidota* and *Firmicutes* (Figure 4E). At the genus level, heat stress reduced the relative abundance of *Bacteroides*, *Lactobacillus*, and *Alistipes*, and increased the relative abundance of *Fusobacterium*, *Thiobacillus*, and *PHOS-HE36* (Figure 4F).

#### 3.4.3. Analysis of significantly different microbial communities

**Figure 5:**
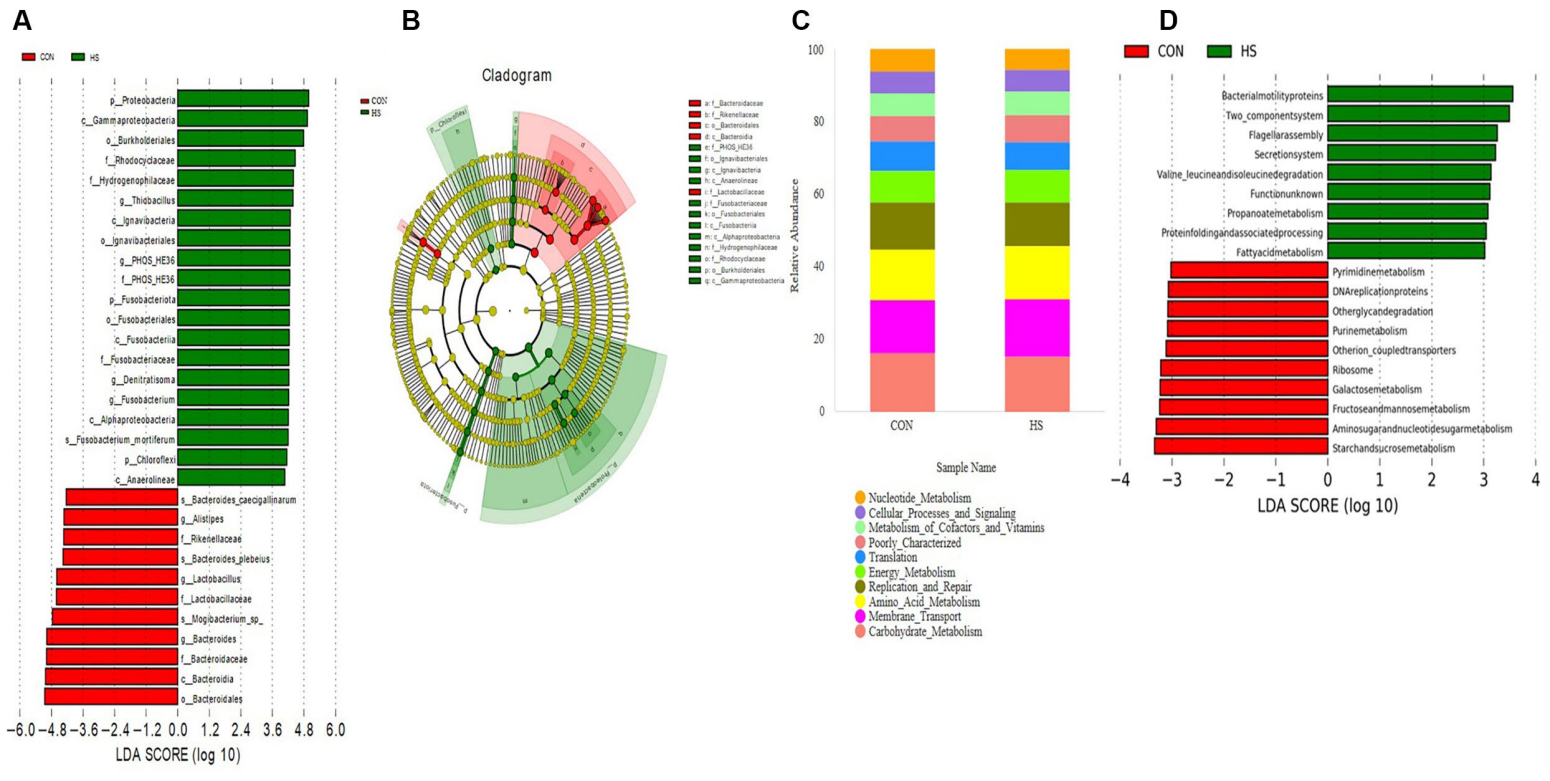
LEfSe analysis and PICRUSt functional prediction. **(A)** LDA bar chart, the length of the bar chart represents the impact of different species, with the LDA = 4. **(B)** Evolutionary branch chart, each small circle at different classification levels represents a classification at that level, and the diameter of the small circle is proportional to the relative abundance. Species with no significant differences are uniformly colored in yellow, and differential species are colored according to the group. **(C)** PICRUSt function predicts the top 10 stacking maps at the level 2. **(D)** PICRUSt function prediction at the level 2 (LDA = 3). The cecal contents of 42-day-old broilers was collected for metabolomic analysis (*n* = 4). CON: broilers were raised in an environment of 21 ± 1°C. HS: broilers were raised in an environment of 33 ± 1°C (9:00–17:00) at 28 days, subjected to heat stress treatment for 8 h, and remained at an appropriate temperature (21 ± 1°C) for the other time as in CON.

Analyze microorganisms with statistical differences between groups using the LEfSe software. Figure 5A shows 31 biomarkers at different classification levels in the CON and HS. Within the HS group of *p_Proteobacteria*, *p_Fusobacteriota*, and *o_Burkholderiales* were significantly higher than the CON. In contrast, the levels of the *g_Alistipes*, *f_Rikenellaceae*, *g_Lactobacillus*, and *g_Bacteroides* were significantly lower than those of the CON. The evolutionary branch indicates (Figure 5B) that the significant differences in microorganisms in the HS are mainly concentrated in *p_Proteobacteria*, *p_Fusobacteriota*, *p_Chloroflexi*. In contrast, the CON mainly concentrates on *c_Bacteroidales* and *f_Lactobacillaceae*.

#### 3.4.4. Functional analysis of cecum microorganisms

The functional prediction of the cecal microbiota is conducted on the PICRUSt platform. By comparing to the KEGG database at level 2 (Figure 5C), the gut microbiota of AA broilers is mainly involved in carbohydrate metabolism, membrane transport, amino acid metabolism, nucleotide metabolism, translation, replication and repair, energy metabolism, metabolism of cofactors and vitamins, poorly characterized, cellular processes and signaling. To further search for differential metabolic pathways, using LEfSe analysis, set LDA = 3. As shown in Figure 5D, the KEGG database at level 3 shows that HS microorganisms are mainly concentrated in fatty acid metabolism, while CON microorganisms are concentrated in such as purine metabolism.

### 3.5. Multi-omics analysis

**Figure 6:**
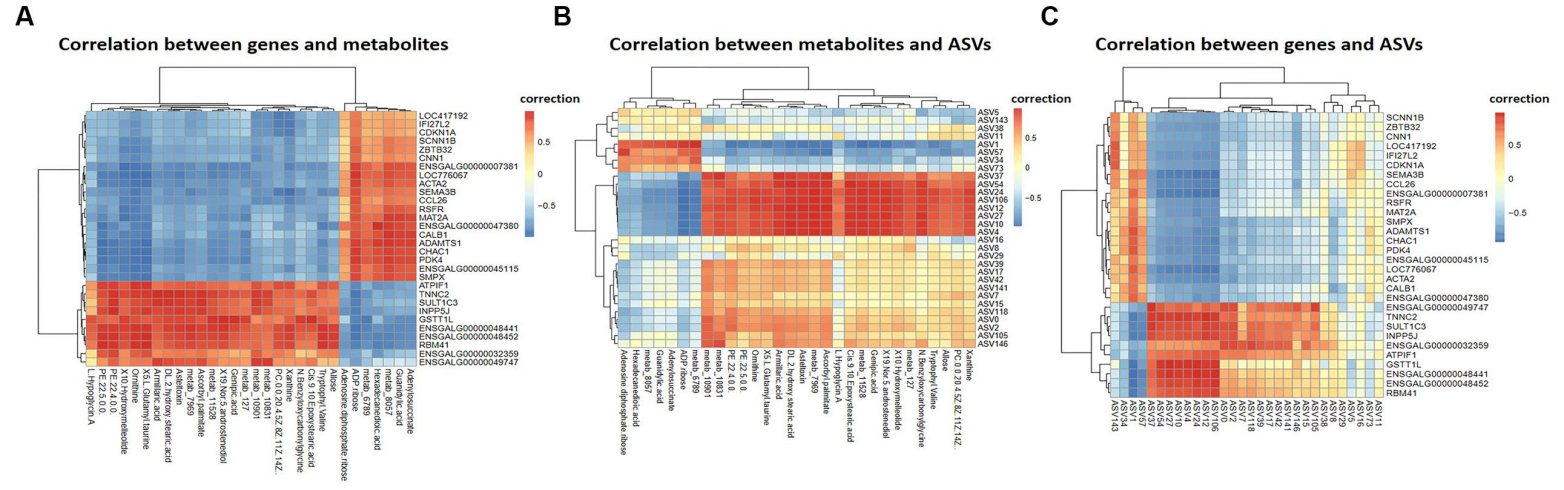
Multi-omics Analysis. **(A)** Differential genes and metabolites correlation heatmap, showing the correlation between top 50 differential genes and metabolites, **(B)** differential metabolites and ASVs correlation heatmap, **(C)** differential genes and ASVs correlation heatmap. Differential genes, differential metabolites and ASVs were derived from cecal transcriptome data (*n* = 3), cecal metabolome data (*n* = 6) and 16 s rDNA amplification data (*n* = 4) of 42-day-old broilers, respectively.

**Figure 7:**
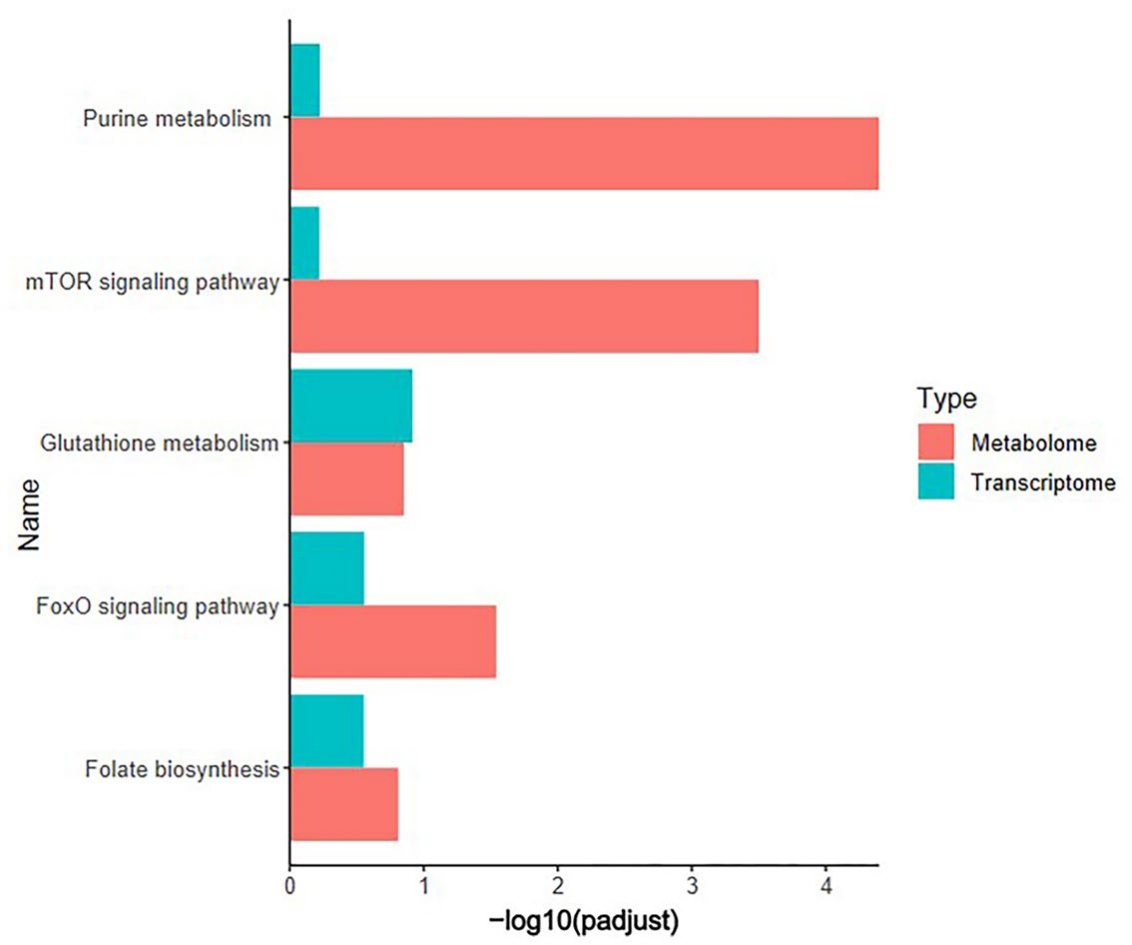
KEGG co-enrichment analysis. Transcriptome (*n* = 3) and metabolome (*n* = 6) co-enrichment pathways in the cecum of 42-day-old broilers.

Pearson correlation analysis was conducted on differential genes and metabolites, differential metabolites and ASVs, and differential genes and ASVs. As shown in Figures 6A,B, based on the differential metabolites detected in the HS, ornithine was positively correlated with *SULT1C3* (correlation = 0.873, Log2 Foldchange = −1.144, *P*adjust < 0.001), *GSTT1L* (correlation = 0.853, Log2 Foldchange = −1.127, *P*adjust < 0.01) and ASV2, and negatively correlated with *CALB1*(correlation = −0.907, Log2 Foldchange = 2.251, *P*adjust < 0.001). The differential genes detected in the HS showed a negative correlation between *CHAC1*(correlation = −0.832, Log2 Foldchange = 1.734, *P*adjust < 0.001) and ASV27 (Figure 6C). Phosphatidylethanolamine (PE) was negatively correlated with *CALB1* and *CHAC1*, and positively with ASV27. Further analysis of KEGG co-enrichment in transcriptome and metabolome showed that 5 pathways were enriched (Supplementary Table S9; Figure 7), which has included purine metabolism, mTOR signaling pathway, glutathione metabolism, FoxO signaling pathway and folate biosynthesis. It can be seen from Figures 3D, 5D that the pathway in which KEGG from metabolome and microbiome co-participation is purine metabolism.

## 4. Discussion

As an important economic indicator of meat and poultry, the quality and performance of poultry meat often directly determine the level of breeding efficiency in production, and with the development of the economy, people are increasingly favoring chicken with good taste, good meat quality and richer nutrition (Ma et al., 2022). Poultry exposure to heat stress can disrupt thermoregulation and homeostasis, reduce poultry performance, health and welfare, and lead to weight loss or even negative growth, low immunity, and even death (Mujahid et al., 2009; Kim et al., 2021; Sarsour et al., 2022). Growth performance is an important indicator for evaluating whether the production efficiency of poultry meets people’s expectations. The decrease in growth performance caused by heat stress is related to decreased food intake (Peng et al., 2023). The result of this study indicated that heat stress reduced the weight and ADG of broilers but had no effect on ADFI and FCR. The results of the current study are inconsistent with previous studies on ADFI and FCR. Previous studies have found that high-temperature environments reduced broilers’ body weight, ADG, and ADFI (Deng et al., 2023). Sun S. et al. (2023) conducted heat stress treatment on 28-day-old broilers and found a significant reduction in ADFI, ADG, and FCR in 42-day-old broilers. In addition, Yilmaz and Gul (2023) found that heat stress reduced the ADG in 42-day-old broilers, but had no significant effect on the FCR. The above results indicate that this study demonstrates the negative effects of heat stress on broilers, indicating that a heat stress model has been successfully established. This may be because when the temperature recovers, periodic heat stress can lead to birds overeating, thereby weakening the impact of heat stress on ADFI. In addition, the impact of heat stress on the growth performance of broilers not only depends on feed intake, but also includes other factors such as physiological, biochemical, hormonal changes, breeds of broiler, duration of heat stress, and temperature of heat stress treatment (Al-Abdullatif and Azzam, 2023). Research has shown that heat stress can damage growth performance by reducing protein and nutrient digestibility; Insulin growth factor in the Endocrine system is the main regulator of muscle metabolism, participating in all stages of muscle formation and muscle regeneration, which can increase protein synthesis and promote differentiation, while heat stress will affect the secretion of insulin growth factor and thus reduce protein synthesis (Nawaz et al., 2021).

Research shows that transcriptome can help researchers analyze which pathways and genes are activated in response to the stressor (Zhang Y. et al., 2023; Zhang F. et al., 2023; Zhang X. et al., 2023). Compared to other parts of the intestine, the cecum of broilers plays a more important role in host defense (Khan and Chousalkar, 2020). Therefore, this is more important for studying the effects of heat stress. From the transcriptome results, 96 differential genes were identified, 78 differential genes of which were up-regulated and 18 differential genes were down-regulated. *PDK4* (pyruvic acid dehydrogenase 4), as a gene with significant differences, plays an important role in regulating energy homeostasis metabolism, glycolysis, and fat decomposition (Honda et al., 2017; Wen et al., 2021; Forteza et al., 2023). In mice with ischemic stroke, it was found that the cecum metabolism was disordered, and the *PDK4* related to fatty acid metabolism was up-regulated, which may lead to the reduction of steroid metabolic process activity (Ge et al., 2022). Research has shown that *PDK4* was previously identified as a differential gene in multiple chicken heat stress experiments (Wang et al., 2021). In the experiment on high-altitude-stressed Tibetan sheep, glycolysis can increase ATP content by up-regulating the expression of *PDK4*, providing energy for resisting hypoxia stress (Wen et al., 2021). As is well known, high temperatures can damage protein stability and lead to dysfunctional cell function (Mackei et al., 2021). *FKBP10* is a member of the FK113 binding protein gene family and is involved in many functions, including protein folding and repair in response to heat stress, which is necessary to maintain natural peptides and prevent protein aggregation (Akbarzadeh et al., 2018). Our previous research found that heat stress can affect lipid metabolism and increase cholesterol content in broilers (Zhang L. et al., 2022). Studies have found that LBP (lipopolysaccharide binding protein) has been used as an indicator of the impairment of intestinal barrier function, and its level can reflect the degree of intestinal leakage (Vancamelbeke and Vermeire, 2017; Wu et al., 2023). Intestinal barrier dysfunction can further contribute to the occurrence of alcoholic hepatitis by acting on the gut-liver axis, triggering inflammatory cascade reactions, and aggravating liver inflammation and LBP levels (Tilg et al., 2016). Heat stress can lead to an increase in LBP levels, which is consistent with our trend (Wickramasinghe et al., 2023). GO results indicated that differential genes were mainly enriched in immune processes. The KEGG results further indicated that differential genes were mainly enriched in other pathways, such as the NF-kB signaling pathway, calcium signaling pathway, and intestinal immune network for IgA production. These pathways play an important role in the intestinal injury. It is reported that heat stress can result in gut microflora dysbiosis, cause bacterial translocation, and thus induce the production of intestinal endotoxin. These endotoxins can activate TLR4-mediated reactions, including initiation of the NF-κB signaling pathway (Tang et al., 2021). NF-κB is a major transcription factor involved in inflammatory diseases, which can respond to heat-stress stimuli and activate the NF-κB signaling pathway in broilers (Xu et al., 2023), thereby inducing tissue damage (Liu W. C. et al., 2021; Liu Y. R. et al., 2021). NF-κB acts downstream of TLR4 and other immune receptors, increasing the excessive production of IL-6, IL-1β, and TNF-α leads to the occurrence of inflammatory responses in response to heat stress (Vallabhapurapu and Karin, 2009). Liu et al. (2022, 2022a,b) found that chronic heat stress can enhance the NF-κB signaling pathway and promote the occurrence of liver inflammation in broilers. The intestinal immune network for IgA production have been confirmed to play an important role in immunity (Zhang et al., 2018). The intestines are the largest lymphoid tissue, and intestinal immunity can produce many non-inflammatory IgA antibodies, the first line of defense against heat stress. Multiple cytokines (*TGF-β*, *IL-10*, *IL-4*, *IL-5* and *IL-6*) are essential for B cells to differentiate into IgA plasma cells (Nagatake et al., 2019; Yang et al., 2021). IgA primarily functions in the intestinal cavity through secretory immunoglobulin A (SIgA), which maintain intestinal mucosal homeostasis and prevent harmful substances from adhering and entering the intestinal barrier (Lammers et al., 2010). Research has shown that chronic heat stress can damage intestinal immune function by promoting the inflammatory response and reducing IgA secretion (Yang et al., 2019). Heat stress can also affect the calcium signaling pathway, leading to mitochondrial oxidative stress and severe calcium overload, damaging mitochondrial structure and function, and even leading to apoptosis (Coble et al., 2014; Zhang W. et al., 2022; Yao et al., 2023).

Metabolomics research can understand changes in the metabolism of organisms, helping researchers better understand how chicken products are affected by the external environment (Zhang Y. et al., 2023; Zhang F. et al., 2023; Zhang X. et al., 2023). The fermentation products produced by cecal microorganisms have a positive impact on intestinal health, and numerous cecal metabolites play a crucial role in maintaining intestinal barrier function (Liu et al., 2022, 2022a,b). Therefore, this is more important for studying the effects of heat stress on changes in cecal metabolites in broilers. According to the metabolomics results, 619 differential metabolites were identified, with 47 metabolites known structures, of which 13 metabolites were up-regulated and 34 were down-regulated. As one of the most differential metabolites of metabolome, ascorbyl palmitate has the function of clearing reactive oxygen species (ROS) and protecting DNA damage when coping with stress, and plays a key role in protecting cells and cell membranes from oxidative damage (Xiao et al., 2014). In addition, ascorbyl palmitate is believed that increasing the amount of Vitamin C will increase the resistance to oxidative stress, thus increasing the resistance to certain diseases (Nieva-Echevarría et al., 2021). Therefore, the oxidative damage caused by heat stress also reflects the reduction of ascorbyl palmitate content. Zhang Y. et al. (2023), Zhang F. et al. (2023), and Zhang X. et al. (2023) showed that the increase of ADP ribose content can identify damaged DNA and further activate the base excision repair mechanism to participate in heat stress regulation. This also confirms the upregulation of ADP ribose metabolite content in this study. KEGG enrichment results showed that differential metabolites were mainly enriched in the purine metabolism and ABC transporters. Purine is involved in DNA and RNA functions and is essential for cell survival and proliferation (Pedley and Benkovic, 2017). Heat stress will lead to oxidative stress in organisms, and purine metabolism is the basic reaction of oxidative stress and the imbalance between purine remedy and *de novo* synthesis pathway will lead to the production of ROS (Yu et al., 2021; Tian et al., 2022). Besides, the metabolism of intestinal microorganisms will affect the content change of metabolites. In this study, the differential pathway of microbial function prediction also includes purine metabolism, which also explains why it is enriched in metabolome data. ABC transporter is a transmembrane protein that can transport many molecules across the cell membrane (Yu et al., 2021). As a member of the ABC transporters, ABCB10 deficiency may lead to mitochondrial oxidative damage and ROS production (Liesa et al., 2012). ROS exceeding the tolerance threshold level of the body can damage the body’s antioxidant system, leading to a decrease in antioxidant enzyme activity (Rahman and Rahman, 2012).

Microorganisms living in the gastrointestinal tract can affect poultry’s nutrition, physiology and intestinal development (Shang et al., 2018). Research has shown that heat stress can increase microbial communities’ richness and abundance indicators, increase OUTs (Operational taxonomic units), and thus increase the Chao1, Shannon, Simpson, and ACE indexes (Goel et al., 2022a; Liu et al., 2022, 2022a,b). This is consistent with the results of this study, which found that heat stress increased the number of OTUs and α diversity index, which may be due to an increase in harmful microbial communities. At the phylum level, heat stress increased the relative abundance of *Proteobacteria* and decreased the relative abundance of *Bacteroidota* and *Firmicutes*. At the genus level, heat stress reduced the relative abundance of the *Alistipes* and increased the relative abundance of the *Fusobacterium*. LEfSe analysis showed that *p_Proteobacteria* and *p_Fusobacteriota* were the differential microbiota under heat stress. At the same time, the relative abundance of *g_Alistipes* and *f_Rikenellaceae* was significantly lower than that of the CON. Functional prediction analysis at levle 2 showed that the gut microbiota of AA broilers was mainly involved in carbohydrate metabolism, membrane transport, amino acid metabolism, replication and repair. This is consistent with the Li et al. report’s functional prediction results (Yi et al., 2023). At the level 3 KEGG metabolic pathway showed that fatty acid metabolism was enhanced in the HS. Research has shown that fatty acid metabolism is an important mechanism closely related to energy homeostasis under heat stress (Lim et al., 2022). This is similar to the report of Jastrebski et al. (2017) that heat stress can increase the expression of enzymes related to fat metabolism, thereby improving fatty acid metabolism. In addition, Goel et al. (2022b) research found that heat stress can reduce the number of *Bacteroidetes* in the ileum of broilers. *Bacteroidetes* are generally considered to maintain complex and beneficial relationships with the host, including fermenting carbohydrates to produce volatile fatty acids (VFAs) as an energy source for host utilization and are positively correlated with growth performance (Zhu et al., 2021). In addition, *Firmicutes* and *Proteobacteria* are related to the fermentation of undigested dietary components. *Firmicutes* contribute to the production of the polysaccharide and butyrate (Wu et al., 2018). Notably, a high proportion of *Proteobacteria* indicates intestinal ecological imbalance and is associated with the pathogenesis of many diseases, such as diarrhea, inflammatory bowel disease, and colitis (Wu et al., 2021). *Alistipes* belonging to the *Bacteroidota* is considered a relatively new bacterial genus that has protective effects on specific diseases, including liver fibrosis, colitis, cancer immunotherapy and cardiovascular diseases (Parker et al., 2020). It is also a major producer of short chain fatty acids in bacteria (Qi et al., 2019). They have the characteristics of glycolysis and proteolysis and produce acetic acid by producing fibrinolysis, digesting gelatin and fermenting carbohydrates (Zhu et al., 2019). As a member of *Fusobacteriota*, the increase in abundance of *Fusobacterium* can act as a pro-inflammatory factor to promote the occurrence of intestinal tumors (Patra and Kar, 2021). In addition, *Fusobacterium* metabolites may make the tumor microenvironment more comfortable over time by directly promoting tumor cell proliferation, vascular growth or immune cell infiltration (Kostic et al., 2013). Research found that the *Rikenellaceae* was related to metabolism and gastrointestinal health in the body and a large number of *Rikenellaceae* had the potential to protect against cardiovascular and metabolic diseases related to visceral fat and were potential biomarkers of healthy aging and longevity (Pin Viso et al., 2021; Tavella et al., 2021; Wang et al., 2022). Therefore, the reduction of the relative abundance of *Rikenellaceae* may lead to the shortening of cell life, which also confirms that differential genes are enriched in cellular senescence.

There is a strong interdependence between gut microbiota, genes and metabolites. Therefore, multi-omics analysis can help us analyze specific mechanisms of action. Pearson correlation analysis showed that ornithine was negatively correlated with *CALB1* and positively correlated with *SULT1C3*, *GSTT1L* and ASV2 (*g_Lactobacillus*). PE was negatively correlated with *CALB1* and *CHAC1*. Studies have shown that amino acids serve as components of synthetic proteins and as important physiological and behavioral regulators, such as regulating stress responses (Chowdhury et al., 2021). Long-term heat stress can reduce the content of most free amino acids (Chowdhury et al., 2021), such as a decrease in ornithine content in the plasma of laying hens exposed to long-term heat stress (Chowdhury et al., 2014). L-ornithine is one of the metabolites in the urea cycle, proline, glutamate, arginine, and polyamine metabolism. It can stimulate the secretion of growth hormone by the pituitary gland and promote the breakdown and metabolism of proteins, sugars and fats (Xiong et al., 2016). Meanwhile, under heat stress conditions, a decrease in ornithine content may weaken the stimulation of glutathione metabolism, thereby reducing the antioxidant capacity of broilers (Xiong et al., 2016). *SULT1C3*, which has sulfotransferase activity, plays a role in larger biomolecules, including proteins and carbohydrates, and is vital in maintaining tissue structure and cellular signaling (Rondini et al., 2014). *GSTT1L* is believed to be related to glutathione metabolism in broilers, participating in antioxidant activity, improving heat tolerance and aging (Zhang et al., 2017; Liu et al., 2019). However, research has found that supplementing *Lactobacillus* probiotics can improve the antioxidant capacity of broilers (Dashti and Zarebavani, 2021). The above results confirm the findings of this study. Therefore, the downregulation of these genes and microorganisms reflects a decrease in antioxidant levels and heat tolerance in broilers. Heat stress is related to the secretion of many hormones (such as estrogen, glucocorticoid, and catecholamine), which may affect the expression of the *CALB1* in tissues in different ways (Ebeid et al., 2012). CALB1, as an intracellular transporter protein, can transport Ca^2+^ and act as a Ca^2+^ sensor to prevent increased intracellular Ca^2+^ concentration from causing toxicity (O'Toole, 2011). In addition, CALB1 is a rate-limiting step for epithelial cells to absorb calcium, thus CALB1 expression is highly correlated with intestinal calcium absorption efficiency (Valable et al., 2020). Bahadoran et al. (2018) found that heat stress can increase the expression of the *CALB1* in the uterus of laying hens, which helps to increase the resistance of uterine cells to the harmful effects of heat stress. As the main source of phospholipids, PE plays an important role in the integrity of cells and organelle membranes in broilers (Guo et al., 2021b). Research shows that heat stress leads to the decrease of phospholipid level in broilers, which may be due to the damage to cell membrane caused by heat stress by increasing the activity of phospholipase A2 and promoting the decomposition of phospholipids (Guo et al., 2021b). The overexpression of *CHAC1*, which is induced in response to endoplasmic reticulum stress, will lead to large glutathione consumption (Aquilano et al., 2014; He et al., 2021). In addition, the high expression of *CHAC1* in male broilers indicates that the degradation rate of glutathione is higher than average, which may play an important role in oxidative stress (Brothers et al., 2019). This is consistent with our research results. Therefore, the broiler will upregulate the expression of *CALB1* and *CHAC1* by responding to cell damage caused by heat stress, thereby increasing the body’s resistance. Further research has found that KEGG co-enrichment of differential genes and differential metabolites indicated such as folate biosynthesis, the FoxO signaling pathway, and the mTOR signaling pathway. According to reports, folic acid not only participates in nutrient metabolism but also has free radical scavenging and ROS activity. Dietary supplementation with folic acid can improve the antioxidant performance and immune status of broilers under heat stress, which may be due to the role of folic acid in regulating protein metabolism (Gouda et al., 2020). The enriched *ALPL* in folate biosynthesis can involve in inflammatory response, bone growth, and bone calcium metabolism (Sharma et al., 2014). High-temperature environments may lead to inflammatory reactions and disrupt bone calcium metabolism in poultry. Therefore, we speculate that upregulation of the *ALPL* can maintain bone health and reflect the resistance of poultry to high temperatures and pathogens. The mTOR signaling pathway is a key nutrient perception pathway that regulates cell metabolism and lifespan in response to various changes in stress, growth factors, and cell energy levels (Su et al., 2019). A previous study suggested that bovine mammary epithelial cells may resist heat stress damage by enhancing the absorption and metabolism of intracellular amino acids and activating the mTOR signaling pathway (Fu et al., 2021). In addition, heat stress significantly upregulated the expression of mTOR signaling pathway related genes, which is consistent with our research results (Fu et al., 2021). L-arginine, as a metabolite significantly enriched in the mTOR signaling pathway, is essential for maintaining growth, reproduction, and immunity (Murakami et al., 2012). Research has found that supplementing arginine can enhance the development of the small intestine and nutrient absorption (Abdulkarimi et al., 2019). Therefore, the decrease in L-arginine content also reflects that heat stress can cause damage to the body and reduce immune function. The FoxO signaling pathway is involved in various cellular functions, including cell proliferation, apoptosis, autophagy, oxidative stress, and metabolic disorders (Xing et al., 2018). According to reports, under environmental stress conditions, the FoxO signaling pathway in fish is significantly enriched, which is consistent with our research results (Shang et al., 2023; Sun J. et al., 2023). The ROS produced by heat stress can regulate the expression of FoxO at the levels of transcription, protein activation, phosphorylation, and acetylation and overactivation and overexpression of FoxO can lead to the occurrence of various diseases (Liu et al., 2023). In addition, FoxO can also affect the expression of the Bcl-2 protein family, stimulate the expression of death receptor ligands and tumor necrosis factor related apoptosis inducing ligands, and induce cell death through mitochondrial mediated endogenous pathways and death receptor mediated exogenous pathways (Fu and Tindall, 2008).

In summary, our study found that heat stress led to decreased growth performance, intestinal oxidative damage, and antioxidant capacity in broilers, a process related to the complex regulation of genes, metabolites, and microorganisms. This will provide a theoretical reference for the poultry industry to improve the problem of heat stress.

## 5. Conclusion

In this study, heat stress reduced the body weight and ADG, altered the expression of purine metabolism, calcium signaling pathway, intestinal immune network for IgA production and FoxO signaling pathway, and increased the expression of cecal microbiota α diversity index, the relative abundance of *Proteobacteria* and *Alistipes* decreased the relative abundance of *Bacteroidetes* and *Firmicutes*. PICRUSt prediction showed that the cecum microorganisms of AA broilers in HS were mainly enriched in fatty acid metabolism, while those in CON were mainly enriched in the purine metabolism pathway. The multi-omic analysis found that the KEGG co-participating pathway of differential genes, metabolites and microorganisms was the purine metabolism. Pearson correlation analysis showed that ornithine was positively correlated with *SULT1C3*, *GSTT1L* and *g_ Lactobacillus*, and negatively correlated with *CALB1*. L-arginine and PC were negatively correlated with *IFI6* and positively correlated with *SULT1C3*. PE was negatively correlated with *CALB1* and *CHAC1*, and positively with *g_Alistipes*.

## Data availability statement

The datasets presented in this study can be found in online repositories. The names of the repository/repositories and accession number(s) can be found below: NCBI and MetaboLights database - Transcriptome sequencing (PRJNA987758), 16S rDNA sampling sequencing (PRNA972642), LC-MS (MTBLS8078).

## Ethics statement

The animal study was approved by Shanxi Agricultural University Ethics Committee. The study was conducted in accordance with the local legislation and institutional requirements.

## Author contributions

XL, YW, ZW, and LZ contributed to the conception, design, and investigation of the study. YW, ZM, and ZW contributed to the initial statistical analysis and bioinformatics analysis. XL completed the final statistical analysis, bioinformatics analysis, visualization, and wrote the first draft of the manuscript. XL, ZM, YW, HJ, ZW, and LZ contributed to manuscript revision. All authors contributed to the article and approved the submitted version.

## Funding

This study was supported by the Youth Fund Project on Application of Basic Research Project of Shanxi Province (202103021223135), the Key R&D project of Shanxi Province (201803D221023-2), the Excellent doctors come to Shanxi to reward scientific research projects (SXBYKY20222057), and the Cultivate Scientific Research Excellence Programs of Higher Education Institutions in Shanxi (2021BQ114).

## Conflict of interest

The authors declare that the research was conducted in the absence of any commercial or financial relationships that could be construed as a potential conflict of interest.

## Publisher’s note

All claims expressed in this article are solely those of the authors and do not necessarily represent those of their affiliated organizations, or those of the publisher, the editors and the reviewers. Any product that may be evaluated in this article, or claim that may be made by its manufacturer, is not guaranteed or endorsed by the publisher.

## Footnotes

^1^ http://asia.ensembl.org/Gallus_gallus/Info/Index
